# Health Tract, No. 144

**Published:** 1863-06

**Authors:** 


### HOUSEHOLD ECONOMY.

No honest man, no wife of sterling principle, can consistently allow a penny's worth to be wasted, as long as a dollar is owing to any body. It is the sinful and dishonest waste in the kitchen which is the last pound to break the camel's back, as to many a striving husband and father in cities and large towns. There are so-called wives who never enter the cellar or store-room; who never look at bills, to see if there are wrong charges, or if provisions are rising rapidly in price, that they might arrange matters so that the aggregate family expenses shall not be increased. A dozen pounds of meat may appear on the dinner-table to-day, for a family of small children and servants, and to-morrow it is all gone, and they " never noticed it." If coffee was a dollar a pound, they would never be the wiser, unless it was mentioned in some conversation. The general result of such indifference is, that the husbands of more thrifty, more notable wives, have eventually to pay the bills. We often talk glibly about French frivolity; but there is much in their domestic management which entitles them to our respect and appreciation. The Paris correspondent of that excellent paper, the New-York *Commercial Advertiser*, writes:

"There are few American families who know exactly the expenses of a year; they all know, probably, that it costs about so many hundred or thousand dollars on the whole. But every European family knows the expense of every year, month, and day; the exact cost of every dinner, supper, or breakfast, of every morsel they eat, of every drop they drink. Every German or French housewife knows not only how much the meat, potatoes, and bread of any meal may cost, but also the water in which she has cooked them, and the coal or wood she has burned to boil the water.

"In Paris, water is sold by barrels and pailfuls. In a house of five stories there are two families on each floor, making ten who ascend the same staircase, up which all articles for family use must be carried. Water, coal, and all heavy articles must be taken up before noon, as about that time the *concierge* cleans the hall and stairs, and they must be kept clean for callers in the afternoon. In every kitchen is a receptacle for water, containing two or more pailfuls. In one corner of the box is a small portion of porous stone, which serves as a filter, and to which is a separate faucet. The *porteur* brings two large pailfuls of water for three cents, every morning. It is therefore, very easy to know how much the water costs in which the dinner is boiled.

"In the same kitchen is a box for coal, which contains the quantity for which they pay forty cents, and they know exactly how many meals can be cooked with this quantity. If they have guests to dinner, they use an extra quantity of water and coal, and know how many cents' worth are devoted to each guest, and then of course they know if they can afford to invite any body again.

"They know exactly how much of every article is used every day. The streets of Paris are lined with small groceries, where every thing is purchased by the cent's worth, and are certainly very convenient for people who earn only a few cents per day.

"The morning meal in every French family is bread and coffee, what they call *café au lait*, and is made of equal portions of coffee and chickory placed in a biggin, upon which hot water is poured so long as it runs through black. Of this they take two spoonfuls to a half-pint of boiling milk. Three or five cents' worth of coffee is purchased every day, and the milkman and baker of course come every morning.

"The second meal is at noon, though it is called breakfast, and is merely a luncheon, cold, or the remnants of yesterday's dinner. For these two no cloth is put upon the table, and all ceremony is unnecessary.

"The dinner is at six, and consists of meat and one vegetable, and something as a salad. The salad is dressed with oil and vinegar—a spoonful of vinegar to three of oil, with pepper, salt, and mustard, and also a little onion and garlic. The commencement of dinner is of course soup, as this is invaluable in every continental family. There are also soup-shops, where a pint or a quart can be purchased every day, between four and six. But as often as once or twice a week they have a boiled dinner, what they call *pot au feu*. In America, the liquor in which meat and vegetables are boiled for such a dinner is thrown away. It must certainly contain the best juice of the meat, and be very good and nourishing. In Europe it is every drop saved and eaten. They fill an earthen pot with meat and vegetables, never omitting the onions, and let it boil away one half. For the soup, they season it with pepper, and sometimes with sorrel, parsley, and other herbs and spices, and thicken it with vermicelli or crumbs of bread. Whether it is delicious or not, it certainly seems too good to throw away.

"The dessert is almost invariably bread and cheese in winter, with a little comfiture. I do not mean to say that every family lives in this way; but I have been in many, and seen little difference. One is expected to take a bit of cheese about an inch square, and a teaspoonful of comfiture. The little shop-windows are also lined with jars of preserves, which are sold in quantities of two or three cents' worth, like any thing else.

"Cheese in the same way, a bit a few inches square for dinner. The pepper and salt are no exceptions to the three-cent rule, little three-cornered papers being the only receptacles for them. Cinnamon, cloves, nutmeg, and similar spices have no location in a continental family, where they never make a pudding or pie or cake of any description, and where they would consider it the greatest extravagance to eat such things. We are talking of families who have a regular income of six hundred to fifteen hundred dollars a year. Such a family does not allow the whole expense of the table to be more than eight or ten dollars a month each person, and we know those who limit it to five or six, and yet who live very comfortably."

